# Genetic variation in folate metabolism is associated with the risk of conotruncal heart defects in a Chinese population

**DOI:** 10.1186/s12887-018-1266-9

**Published:** 2018-08-30

**Authors:** Xike Wang, Haitao Wei, Ying Tian, Yue Wu, Lei Luo

**Affiliations:** 1 Department of paediatrics, Guizhou Provincial People’s Hospital, Guiyang, 550002 China; 2 Department of science and education, Guizhou Provincial People’s Hospital, Guiyang, 550002 China

**Keywords:** Conotruncal heart defect, Single-nucleotide polymorphism, Methionine synthase, Methylenetetrahydrofolate reductase, Solute carrier family 19

## Abstract

**Background:**

Conotruncal heart defects (CTDs) are a subgroup of congenital heart defects that are considered to be the most common type of birth defect worldwide. Genetic disturbances in folate metabolism may increase the risk of CTDs.

**Methods:**

We evaluated five single-nucleotide polymorphisms (SNPs) in genes related to folic acid metabolism: methylenetetrahydrofolate reductase (MTHFR C677T and A1298C), solute carrier family 19, member 1 (SLC19A1 G80A), methionine synthase (MTR A2576G), and methionine synthase reductase (MTRR A66G), as risk factors for CTDs including various types of malformation, in a total of 193 mothers with CTD-affected offspring and 234 healthy controls in a Chinese population.

**Results:**

Logistic regression analyses revealed that subjects carrying the TT genotype of MTHFR C677T, the C allele of MTHFR A1298C, and the AA genotype of SLC19A1 G80A had significant 2.47-fold (TT vs. CC, OR [95% CI] = 2.47 [1.42–4.32], *p* = 0.009), 2.05–2.20-fold (AC vs. AA, 2.05 [1.28–3.21], *p* = 0.0023; CC vs AA, 2.20 [1.38–3.58], *p* = 0.0011), and 1.68-fold (AA vs. GG, 1.68 [1.02–2.70], *p* = 0.0371) increased risk of CTDs, respectively. Subjects carrying both variant genotypes of MTHFR A1298C and SLC19A1 G80A had a higher (3.23 [1.71–6.02], *p* = 0.0002) increased risk for CTDs. Moreover, the MTHFR C677T, MTHFR A1298C, and MTRR A66G polymorphisms were found to be significantly associated with the risk of certain subtypes of CTD.

**Conclusions:**

Our data suggest that maternal folate-related SNPs might be associated with the risk of CTDs in offspring.

## Background

Congenital heart defects (CHDs) are the most common type of birth defect and are associated with significant morbidity and mortality. CHDs occur in approximately 0.4–1% of children born alive [1, 2]. CHDs include a broad range of different forms of structural malformations that are developmentally and clinically heterogeneous [3, 4]. Among the identified subgroups of CHDs, conotruncal heart defects (CTDs) account for 25–33% of all patients [4]. This CHD subgroup involves cardiac structures that are partially derived from cell lineages [5], and includes malformations such as tetralogy of Fallot (TOF), pulmonary atresia with ventricular septal defect (PA/VSD), double outlet of right ventricle (DORV), transposition of the great arteries (TGA), persistent truncus arteriosus (PTA), and interrupted aortic arch (IAA). CTD was considered to be a folate-sensitive birth defect because women who take multivitamins containing folic acid early in pregnancy are at approximately a 30–40% reduced risk of delivering offspring with these heart defects [6, 7]. Although the protective mechanism of folic acid is unclear, evidence has been reported that genetic variations that alter the activity of key enzymes in the folate pathway could influence the risk of such heart defects [8–10].

Although the folic acid cycle is highly complex in mammals, various genes controlling folate metabolism, such as methylenetetrahydrofolate reductase (MTHFR), solute carrier family 19, member 1 (SLC19A1), methionine synthase (MTR), and methionine synthase reductase (MTRR), have been proven to play crucial roles in this metabolic pathway. For example, the MTHFR gene, located on chromosome 1p36.3, encodes an enzyme that catalyzes the reduction of 5,10-methylenetetrahydrofolate to 5-methyltetrahydrofolate [11], which is essential for folate-mediated one-carbon metabolism. SLC19A1 has also been referred to as reduced folate carrier-1 (RFC1), which is involved in the active transport of 5-methyltetrahydrofolate from the plasma to the cytosol and the regulation of intracellular concentrations of folate [12]. MTR catalyzes the remethylation of homocysteine to methionine [13], while MTRR catalyzes the regeneration of the cobalamin cofactor of MTR, thus maintaining MTR in an active state [14]. A single-nucleotide polymorphism (SNP) is a variation in a single nucleotide that is present to some appreciable degree within a population. Many studies have investigated associations between SNPs in the above-mentioned genes and the risk of CHD/CTD. Among them, the MTHFR C677T variant (TT), MTHFR A1298C variant (CC), SLC19A1 G80A variant (AG or AA), MTR A2576G variant (GG), and MTRR A66G variant (GG) have been extensively investigated. Although these gene variants would theoretically influence the risk of CHD/CTD, studies have yielded conflicting results on this issue in different populations [10, 12, 15–19].

Based on the results of previously published studies, we concluded that polymorphisms in genes that encode these key enzymes in the folate pathway would alter its activity, but there is debate on whether these genetic variants affect the risk of heart defects. In the present study, we thus aimed to determine whether the maternal polymorphisms of MTHFR C677T, MTHFR A1298C, SLC19A1 G80A, MTR A2576G, and MTRR A66G in a Chinese population are associated with various types of CTD.

## Methods

### Patients and controls

The present study was approved by the ethics committee of Guizhou Provincial People’s Hospital. All participants provided written informed consent to approve the use of their blood samples for research purposes. A total of 193 mothers of echocardiographically proven CTD-affected children (CTD group, mean age: 29.4 ± 5.1) and 234 mothers of healthy children (control group, mean age: 29.1 ± 5.1) were recruited in the study between January 2017 and January 2018. All participants were genetically unrelated ethnic Han Chinese. For 193 mothers in the CTD group, each had only one child with CTD, as summarized in Table 1; different types of CTD in the children included TOF (90 cases), PA/VSD (31 cases), DORV (35 cases), TGA (10 cases), PTA (14 cases), and IAA (13 cases). For each mother, 5 ml of peripheral blood was collected in EDTA tubes, and within 5 h, genomic DNA was isolated from whole blood using the QIAamp DNA Blood Mini Kit (QIAGEN, Germany), in accordance with the manufacturer’s protocol. Then, the genomic DNA was either stored at − 80 °C or SNP genotyping was conducted on it immediately.

**Table 1:** Conotruncal heart defects affecting the children

Type of conotruncal heart defect	No. (%)
Tetralogy of Fallot	90 (46.6)
Pulmonary atresia with ventricular septal defect	31 (16.1)
Double outlet of right ventricle	35 (18.1)
Transposition of the great arteries	10 (5.2)
Persistent truncus arteriosus	14 (7.3)
Interrupted aortic arch	13 (6.7)
Total	193

### Polymorphism detection

The polymorphisms of five selected genetic variants were determined by the Taqman SNP Genotyping Assay (Thermo Fisher, USA), Briefly, 50 ng of DNA was amplified using Taqman Genotyping Master Mix (Thermo Fisher, USA) and commercial probes (Thermo Fisher, USA) for MTHFR C677T (rs1801133), MTHFR A1298C (rs1801131), SLC19A1 G80A (rs1051266), MTR A2576G (rs1805087), and MTRR A66G (rs1801394) in a final volume of 25 μL. PCR thermal cycling conditions were as follows: 10 min at 95 °C for AmpliTaq Gold, UP Enzyme activation, and then 40 cycles of denaturation at 95 °C for 15 s and annealing/extension at 65 °C for 1 min.

### Statistical analysis

The statistical analyses were performed using SPSS version 19.0 software. The differences in allele frequencies between patients and controls were evaluated using chi-squared test. The associations between genotypes and the risk of CTD were estimated by calculating the odds ratio (OR) and the 95% confidence interval (CI) from logistic regression analyses.

## Results

### Allele frequencies

The distribution of allele frequencies did not differ for MTR A2576G and MTRR A66G between the CTD and control groups (Table 2). However, statistically significant differences were observed in the distribution of the mutated allele for MTHFR C677T, MTHFR A1298C, and SLC19A1 G80A, in which the frequencies of the T allele (48.7% vs. 38.9%, *p* = 0.004), C allele (52.1% vs. 38.7%, *p* < 0.001), and A allele (46.9% vs. 40.2%, *p* = 0.0485) were higher in the CTD group. These deviations could have been due to genetic associations with CTDs.

**Table 2:** Allele frequencies of the CTD and control groups

Genotyped SNPs	Controls (*n* = 234)	CTD (*N* = 193)	*p*-Value for HWE test
	% (No.)	% (No.)	
MTHFR C677T (rs1801133)			
C	61.1(286)	51.3(198)	0.004*
T	38.9(182)	48.7(188)	
MTHFR A1298C (rs1801131)			
A	61.3(287)	47.9(185)	< 0.001*
C	38.7(181)	52.1(201)	
SLC19A1 G80A (rs1051266)			
G	59.8(280)	53.1(205)	0.0485*
A	40.2(188)	46.9(181)	
MTR A2576G (rs1805087)			
A	41.5(194)	61.9(239)	0.7862
G	58.5(174)	38.1(147)	
MTRR A66G (rs1801394)			
A	56.8(266)	60.6(234)	0.2639
G	43.2(202)	39.4(152)	

*HWE* Hardy-Weinberg equilibrium

*means *p*-value< 0.05

### Association of folate-related SNPs with risk of CTDs

The associations between the risk of CTDs and the homozygous variant genotype, heterozygous variant genotype, and variant allele were evaluated for each of the five folate-related SNPs (Table 3). In the single-locus analyses, the genotype frequencies of MTHFR C677T were 33.68% (CC), 35.23% (CT), and 31.09% (TT) in the CTD group and 35.47% (CC), 51.28% (CT), and 13.25% (TT) in the control group, and the difference was significant for the TT genotype (*p* = 0.0009), when using the CC genotype as a reference point. Logistic regression analyses revealed that subjects carrying the TT genotype had a significant 2.47-fold (OR: 2.47, 95% CI: 1.42–4.32) increased risk of CTDs, compared with the subjects carrying the CC genotype. Moreover, subjects carrying the C allele of MTHFR A1298C had a significant 2.05–2.20-fold increased risk of CTDs (AC vs. AA, OR: 2.05, 95% CI: 1.28–3.21, *p* = 0.0023; CC vs. AA, OR: 2.20, 95% CI: 1.38–3.58, *p* = 0.0011). There was also a significantly higher frequency of the AA genotype for SLC19A1 G80A in the CTD group than in the controls (OR: 1.68, 95% CI: 1.02–2.70, *p* = 0.0371), when using the GG genotype as a reference. However, none of MTR A2576G and MTRR A66G exhibited a statistically significant difference in the genotype distributions between the two groups.

**Table 3:** Genotype frequencies among controls and CTD cases

Genotype	Controls (n = 234)	CTD (N = 193)		*p*-Value
	No. (%)	No. (%)	OR (95% CI)	
MTHFR C677T				
CC	83(35.47)	65(33.68)	1.00	
CT	120(51.28)	68(35.23)	0.72(0.47–1.11)	0.1493
TT	31(13.25)	60(31.09)	2.47(1.42–4.32)	0.0009*
CT + TT	151(64.53)	128(66.32)	1.08(0.73–1.62)	0.6987
MTHFR A1298C				
AA	110(47.01)	57(29.53)	1.00	
AC	67(28.63)	71(36.79)	2.05(1.28–3.21)	0.0023*
CC	57(24.36)	65(33.68)	2.20(1.38–3.58)	0.0011*
AC + CC	124(52.99)	136(70.47)	2.12(1.40–3.19)	0.0002*
SLC19A1 G80A				
GG	102(43.59)	68(35.23)	1.00	
AG	82(35.04)	69(35.75)	1.26(0.82–1.96)	0.3031
AA	50(21.37)	56(29.02)	1.68(1.02–2.70)	0.0371*
AG + AA	132(56.41)	125(64.77)	1.42(0.96–2.09)	0.0791
MTR A2756G				
AA	87(37.18)	66(34.20)	1.00	
AG	120(51.28)	107(55.44)	1.18(0.77–1.76)	0.4426
GG	27(11.54)	20(10.36)	0.98(0.51–1.92)	0.9436
AG + GG	147(62.82)	127(65.80)	1.14(0.77–1.70)	0.5224
MTRR A66G				
AA	75(32.05)	71(36.79)	1.00	
AG	116(49.57)	92(47.67)	0.84(0.55–1.28)	0.4136
GG	43(18.38)	30(15.54)	0.74(0.42–1.32)	0.2917
AG + GG	159(67.95)	122(63.21)	0.81(0.54–1.21)	0.3045

*OR* odds ratio, *CI* confidence interval

*means *p*-value< 0.05

### Association of folate-related SNPs with risk of TOF, PA/VSD, DORV, TGA, PTA, and IAA

We also performed stratification analyses to evaluate the effects of five folate-related SNPs on certain types of CTD (Table 4). Our results suggest that subjects carrying the TT genotype of MTHFR C677T had significantly increased risks of TOF (OR: 2.33, 95% CI: 1.18–4.39, *p* = 0.0111), DORV (OR: 3.87, 95% CI: 1.55–9.32, *p* = 0.0034), and IAA (OR: 4.02, 95% CI: 1.09–13.12, *p* = 0.0297). The C allele of MTHFR A1298C was also associated with an increased risk of TOF (AC vs. AA, OR: 2.01, 95% CI: 1.11–3.70, *p* = 0.0201; CC vs. AA, OR: 2.14, 95% CI: 1.14–3.88, *p* = 0.0133), while it was only statistically significant in homozygote comparisons for DORV (OR: 2.51, 95% CI: 1.00–6.13, *p* = 0.0369) and IAA (OR: 6.75, 95% CI: 1.41–32.67, *p* = 0.008). Moreover, the GG genotype of MTRR A66G was associated with significantly decreased risks of TOF (OR: 0.39, 95% CI: 0.17–0.88, *p* = 0.026) and PA/VSD (OR: 0.12, 95% CI: 0.01–0.71, *p* = 0.021). In addition, subjects carrying the AC genotype of MTHFR A1298C had a significant 3.83-fold increased risk of PTA (OR: 3.83, 95% CI: 1.00–13.9, *p* = 0.0431). However, none of the folate-related SNPs was found to be associated with the risk of TGA.

**Table 4:** Genotype frequencies among controls, TOF, PA/VSD, DORV, TGA, PTA and IAA cases

Genotype	Controls (n = 234)	TOF (*n* = 90)		*p*-Value	PA/VSD (*n* = 31)		*p*-Value	DORV (*n* = 35)		*p*-Value	TGA (*n* = 10)		*p*-Value	PTA (*n* = 14)		*p*-Value	IAA (*n* = 13)		*p*-Value
	No. (%)	No. (%)	OR (95% CI)		No. (%)	OR (95% CI)		No. (%)	OR (95% CI)		No. (%)	OR (95% CI)		No. (%)	OR (95% CI)		No. (%)	OR (95% CI)	
MTHFR C677T																			
CC	83(35.47)	31(34.44)	1.00		13(41.94)	1.00		9(25.71)	1.00		2(20.00)	1.00		6(42.86)	1.00		4(30.77)	1.00	
CT	120(51.28)	32(35.56)	0.71(0.41–1.24)	0.2347	11(35.48)	0.59(0.26–1.39)	0.2130	13(37.14)	0.99(0.40–2.39)	0.9984	5(50.00)	1.73(0.36–8.84)	0.5140	4(28.57)	0.46(0.14–1.80)	0.2315	3(23.08)	0.52(0.13–1.98)	0.3906
TT	31(13.25)	27(30.00)	2.33(1.18–4.39)	0.0111*	7(22.58)	1.44(0.56–3.90)	0.4749	13(37.14)	3.87(1.55–9.32)	0.0034*	3(30.00)	4.02(0.78–23.14)	0.1120	4(28.57)	1.79(0.54–6.38)	0.3883	6(46.15)	4.02(1.09–13.12)	0.0297*
CT + TT	151(64.53)	59(65.56)	1.05(0.64–1.72)	0.8625	18(58.06)	0.76(0.36–1.59)	0.4816	26(74.29)	1.59(0.70–3.60)	0.2565	8(80.00)	2.20(0.52–10.46)	0.3147	8(57.14)	0.73(0.24–2.21)	0.5757	9(69.23)	1.24(0.38–3.72)	0.7298
MTHFR A1298C																			
AA	110(47.01)	27(30.00)	1.00		11(35.48)	1.00		10(28.57)	1.00		4(40.00)	1.00		3(21.43)	1.00		2(15.38)	1.00	
AC	67(28.63)	33(36.67)	2.01(1.11–3.70)	0.0201*	13(41.94)	1.94(0.80–4.39)	0.1255	12(34.29)	1.97(0.85–4.87)	0.1313	2(20.00)	0.82(0.15–3.61)	0.8222	7(50.00)	3.83(1.00–13.90)	0.0431*	4(30.77)	3.28(0.74–17.52)	0.1543
CC	57(24.36)	30(33.33)	2.14(1.14–3.88)	0.0133*	7(22.58)	1.23(0.47–3.39)	0.6869	13(37.14)	2.51(1.00–6.13)	0.0369*	4(40.00)	1.93(0.54–6.80)	0.3575	4(28.57)	2.57(0.67–10.43)	0.2113	7(53.85)	6.75(1.41–32.67)	0.008*
AC + CC	124(52.99)	63(70.00)	2.07(1.22–3.50)	0.0055*	20(64.52)	1.61(0.76–3.63)	0.2261	25(71.43)	2.22(1.02–4.61)	0.0407*	6(60.00)	1.33(0.35–4.26)	0.6635	11(78.57)	3.25(0.97–11.09)	0.0619	11(84.62)	4.88(1.15–22.36)	0.0258*
SLC19A1 G80A																			
GG	102(43.59)	29(32.22)	1.00		13(41.94)	1.00		15(42.86)	1.00		3(30.00)	1.00		3(21.43)	1.00		5(38.46)	1.00	
AG	82(35.04)	35(38.89)	1.62(0.93–2.87)	0.0987	12(38.71)	1.24(0.51–2.90)	0.6163	10(28.57)	0.89(0.38–2.06)	0.7983	1(10.00)	0.45(0.03–3.06)	0.4786	5(35.71)	2.24(0.57–8.62)	0.2691	6(46.15)	1.61(0.45–4.84)	0.4416
AA	50(21.37)	26(28.89)	1.63(0.88–2.99)	0.1205	6(19.35)	0.84(0.31–2.26)	0.7387	10(28.57)	1.21(0.51–2.85)	0.6593	6(60.00)	3.64(0.96–13.59)	0.0593	6(42.86)	3.64(0.96–13.59)	0.0593	2(15.38)	0.73(0.14–3.58)	0.7094
AG + AA	132(56.41)	61(67.78)	1.63(0.96–2.70)	0.0618	18(58.06)	1.07(0.51–2.22)	0.8614	20(57.14)	1.03(0.50–2.12)	0.9350	7(70.00)	1.80(0.49–6.53)	0.3953	11(78.57)	2.83(0.84–9.67)	0.1031	8(61.54)	1.24(0.38–3.44)	0.7165
MTR A2756G																			
AA	87(37.18)	33(36.67)	1.00		13(41.94)	1.00		10(28.57)	1.00		2(20.00)	1.00		3(21.43)	1.00		5(38.46)	1.00	
AG	120(51.28)	46(51.11)	1.01(0.59–1.70)	0.9686	15(48.39)	0.84(0.39–1.83)	0.6585	21(60.00)	1.52(0.70–3.29)	0.3018	6(60.00)	2.18(0.52–10.77)	0.3373	11(78.57)	2.66(0.78–9.10)	0.1289	8(61.54)	1.16(0.35–3.24)	0.8003
GG	27(11.54)	11(12.22)	1.07(0.50–2.37)	0.8623	3(9.68)	0.74(0.21–2.60)	0.6609	4(11.43)	1.29(0.42–4.40)	0.6871	2(20.00)	3.22(0.48–21.06)	0.2295	0(0.00)	NA	NA	0(0.00)	NA	NA
AG + GG	147(62.82)	57(63.33)	1.02(0.61–1.72)	0.9318	18(58.06)	0.82(0.39–1.71)	0.6077	25(71.43)	1.48(0.67–3.10)	0.3226	8(80.00)	2.37(0.56–11.26)	0.2691	11(78.57)	2.17(0.64–7.42)	0.2338	8(61.54)	0.95(0.29–2.64)	0.9259
MTRR A66G																			
AA	75(32.05)	36(40.00)	1.00		14(45.16)	1.00		12(34.29)	1.00		3(30.00)	1.00		2(14.29)	1.00		4(30.77)	1.00	
AG	116(49.57)	46(51.11)	0.83(0.48–1.38)	0.4747	16(51.61)	0.74(0.33–1.54)	0.4423	11(31.43)	0.59(0.26–1.36)	0.2338	3(30.00)	0.65(0.15–2.83)	0.5966	9(64.29)	0.29(0.73–13.68)	0.1615	7(53.85)	1.13(0.34–3.55)	0.8478
GG	43(18.38)	8(8.89)	0.39(0.17–0.88)	0.026*	1(3.23)	0.12(0.01–0.71)	0.021*	12(34.29)	1.74(0.75–4.03)	0.2139	4(40.00)	2.33(0.60–9.50)	0.2719	3(21.43)	2.62(0.51–15.07)	0.2862	2(15.38)	0.87(0.16–3.88)	0.8773
AG + GG	159(67.95)	54(60.00)	0.71(0.43–1.18)	0.1769	17(54.84)	0.57(0.27–1.19)	0.1464	23(65.71)	0.90(0.42–1.91)	0.7921	7(70.00)	1.10(0.29–4.00)	0.8917	12(85.71)	2.83(0.69–12.93)	0.1629	11(84.62)	1.06(0.44–3.81)	0.9232

*OR* odds ratio, *CI* confidence interval

*means *p*-value< 0.05

### MTHFR C677T, A1298C, and SLC19A1 G80A combined genotype frequencies and risk of CTDs

We investigated the association between three combined genotypes (MTHFR C677T and A1298C, and SLC19A1 G80A) and the risk of CTDs (Table 5). Significant differences were only observed in the combined genotype distributions of MTHFR A1298C and SLC19A1 G80A. Subjects carrying either one variant genotype (OR: 1.9, 95% CI: 1.05–3.4, *p* = 0.0382) or both variant genotypes (OR: 3.23, 95% CI: 1.71–6.02, *p* = 0.0002) of these two folate-related SNPs had a significant 1.9–3.23-fold increased risk of CTDs. Moreover, none of the other comparisons produced significant results.

**Table 5:** Combined genotype frequencies of MTHFR C677T, A1298C and SLC19A1 G80A among controls and CTD cases

Genotype	Controls (n = 234)	CTD (N = 193)		*p*-Value
	No. (%)	No. (%)	OR (95% CI)	
MTHFR C677T and A1298C combinations				
677CC, 1298AA	32(13.68)	23(11.92)	1.00	
677CC, 1298 AC + CC	51(21.79)	42(21.76)	1.15(0.60–2.23)	0.6921
1298AA, 677CT + TT	78(33.33)	34(17.62)	0.61(0.32–1.17)	0.1421
Either one variant genotype	129(55.13)	76(39.38)	0.82(0.44–1.49)	0.5199
Both variant genotypes	73(31.20)	94(48.70)	1.79(0.95–3.33)	0.0623
MTHFR C677T and SLC19A1 G80A combinations				
677CC, 80GG	35(14.96)	21(10.88)	1.00	
677CC, 80AG + AA	48(20.51)	44(22.80)	1.53(0.76–3.03)	0.2196
80GG, 677CT + TT	67(28.63)	47(24.35)	1.17(0.60–2.23)	0.6410
Either one variant genotype	115(49.15)	91(47.15)	1.32(0.72–2.44)	0.3706
Both variant genotypes	84(35.90)	81(41.97)	1.61(0.85–3.05)	0.1327
MTHFR A1298C and SLC19A1 G80A combinations				
1298AA, 80GG	46(19.66)	18(9.33)	1.00	
1298AA, 80AG + AA	64(27.35)	39(20.21)	1.56(0.79–3.14)	0.1969
80GG, 1298 AC + CC	56(23.93)	50(25.91)	2.28(1.18–4.53)	0.0141*
Either one variant genotype	120(51.28)	89(46.11)	1.90(1.05–3.40)	0.0382*
Both variant genotypes	68(29.06)	86(44.56)	3.23(1.71–6.02)	0.0002*
MTHFR C677T, A1298C and SLC19A1 G80A combinations				
677CC, 1298AA, 80GG	12(5.13)	10(5.18)	1.00	
677CC, 1298AA, 80AG + AA	20(8.55)	11(5.70)	0.66(0.20–2.09)	0.4646
677CC, 80GG, 1298 AC + CC	23(9.83)	11(5.70)	0.57(0.18–1.77)	0.3226
1298AA, 80GG, 677CT + TT	34(14.53)	10(5.18)	0.35(0.11–1.06)	0.0582
Either one variant genotype	77(32.91)	32(16.58)	0.50(0.20–1.34)	0.1400
677CC, 1298 AC + CC, 80AG + AA	28(11.97)	31(16.06)	1.33(0.48–3.32)	0.5704
677CT + TT, 1298AA, 80AG + AA	44(18.80)	26(13.47)	0.71(0.26–1.78)	0.4859
677CT + TT, 1298 AC + CC, 80GG	33(14.10)	39(20.21)	1.42(0.54–3.52)	0.4740
Either two variant genotypes	105(44.87)	96(49.74)	1.10(0.48–2.67)	0.8370
All variant genotypes	40(17.09)	55(28.50)	1.65(0.66–4.40)	0.2900

*OR* odds ratio, *CI* confidence interval

*means *p*-value< 0.05

## Discussion

Folate is known to play a crucial role in preventing birth defects during pregnancy, including CHD [20]. Thus, genetic variations in components of the folate pathway could influence the risk of CHD. However, the results of studies on the association between folate-related gene polymorphisms and CHD risk are inconclusive and contradictory [9, 12, 17–19]. It was hypothesized that these gene variants may be only associated with specific subsets of CHD, leading to conflicting results when study samples included heterogeneous disease phenotypes [10]. CTDs are the most prevalent congenital anomalies, accounting for approximately one-third of all CHDs, and they play a significant role in fetal morbidity and mortality. To the best of our knowledge, the present study is the first to provide reliable evidence about the association between folate-related gene polymorphisms and the risk of CTDs, specifically including various subtypes of CTD in a Chinese population. This study particularly focused on the maternal genotype. Maternal genetic effects behave as environmental risk factors for offspring [21]. However, it is easier to identify the maternal genotype during pregnancy, so it would be more convenient to translate this approach into a clinical context. For women carrying high-risk genotypes, clinicians could suggest targeted risk reduction strategies aimed at increasing folic acid supplementation.

In this hospital-based case–control study, we analyzed the involvement of five gene variants (MTHFR C677T, MTHFR A1298C, SLC19A1 G80A, MTR A2576G, and MTRR A66G) related to the metabolism of folic acid as risk factors for CTDs. Our results demonstrated that genotypes for MTHFR C677T, MTHFR A1298C, and SLC19A1 G80A might be associated with the risk of CTDs. For certain types of CTD, the genotypes of MTHFR C677T and MTHFR A1298C were also found to be associated with the risks of TOF, DORV, PTA, and IAA, and the GG genotype of MTRR A66G was associated with decreased risks of TOF and PA/VSD.

Because the MTHFR gene plays a key role in folate metabolism through affecting global DNA methylation, which is essential for embryonic development and the formation of the cardiovascular system [22], it has attracted the most attention as an etiological factor for CHDs. Although many studies have indicated that MTHFR C677T and MTHFR A1298C are not strongly related to the risk of CHDs [18, 23, 24], in two recent meta-analyses, Li et al. evaluated 19 eligible studies concerning the MTHFR C677T polymorphism and CHD, comprising 4219 cases and 20,123 controls. They found a significant association between the MTHFR C677T polymorphism and CHD risk in the maternal analysis (OR: 1.52, 95% CI: 1.09–2.11, *p* = 0.01) [25]. In another study by Yu et al., 16 eligible studies concerning MTHFR A1298C polymorphism and CHD, involving 2207 cases and 2364 controls, were included in the meta-analysis; the results suggested that the CC genotype of MTHFR A1298C is a risk factor for CHDs [26]. As well as these previous studies, our results demonstrated that the MTHFR C677T and MTHFR A1298C polymorphisms are also strongly related to the risks of CTDs and of certain types of CTD, including TOF, DORV, PTA, and IAA.

Regarding the MTR and MTRR genes, which play key roles in the second step of folate metabolism and may confer protective effects against CHDs, a recent meta-analysis has also evaluated the associations of MTR A2576G and MTRR A66G polymorphisms with the risk of CHDs. Cai et al. evaluated nine eligible studies comprising 914 cases and 964 controls [27]. The results showed that the MTRR 66G allele significantly increased the risk of CHDs compared with the MTRR 66A allele (OR: 1.35, 95% CI: 1.14–1.59, *p* < 0.001), but no significant differences were found in the MTR A2576G polymorphism between the groups. However, the present results indicate that the allele frequencies of MTR A2576G and MTRR A66G did not differ between the CTD and control groups, except for the MTRR A66G polymorphism, for which the frequency of the GG genotype was significantly lower in the TOF and PA/VSD groups. Moreover, the number of studies focusing on the association of the SLC19A1 G80A polymorphism with the risk of CHDs is small, and the reported results are disputable. For example, Koshy et al. demonstrated that the SLC19A1 G80A polymorphism is not significantly associated with the risk of CTDs in an Indian population [17]. However, Christensen et al. reported that the AG and GG genotypes were associated with decreased odds ratios for heart defects in a Canadian population [28]. By contrast, Gong et al. found that the AG genotype was associated with a significantly increased risk of CHD in a Han Chinese population [10]. As well as the present results on MTR A2576G and MTRR A66G polymorphisms being the opposite of those of several studies concerning CHDs, our results show that the AA genotype of SLC19A1 G80A is associated with a significantly increased risk of CTDs, which also differs from the finding of the previous study by Gong et al. These discrepancies might have arisen because the study samples included different disease phenotypes. Otherwise, the subjects exhibited differences in the regular intake of folic acid because the gene polymorphisms might influence the risk of CTDs only in situations in which the intake of folic acid is insufficient. However, further studies on these issues are required. In addition, we also found a significant genotype interaction between MTHFR A1298C and SLC19A1 G80A. Mothers carrying both variant genotypes of these two SNPs had a higher increased risk for CTDs compared with mothers carrying single variant genotypes. The mechanism linking these factors remains unclear, so further studies of this issue are also required.

The present study had several limitations. First, it was a hospital-based case–control study, so the recruited subjects may not be representative of the general population. Second, there was a lack of information on maternal folate status, so we could not determine whether the gene polymorphisms could influence the risk of CTDs if sufficient folic acid were consumed, and whether this variable was a cause of the heterogeneity of the results among different studies. Third, the sample size was moderate in this study, and in the subgroup analyses including PA/VSD, DORV, TGA, PTA, and IAA there were relatively small numbers of cases in each group. Therefore, further studies with larger sample sizes are required to confirm the present findings.

## Conclusions

Our results demonstrated that maternal genotypes of MTHFR C677T, MTHFR A1298C, and SLC19A1 G80A might be associated with the risk of CTDs. In addition, the maternal genotypes for MTHFR C677T, MTHFR A1298C, and MTRR A66G might be associated with the risk of certain types of CTD, including TOF, PA/VSD, DORV, PTA, and IAA.

## Abbreviations

CHD: Congenital heart defect
CTD: Conotruncal heart defect
DORV: Double outlet of right ventricle
IAA: Interrupted aortic arch.
MTHFR: Methylenetetrahydrofolate reductase
MTR: Methionine synthase
MTRR: Methionine synthase reductase
PA/VSD: Pulmonary atresia with ventricular septal defect
PTA: Persistent truncus arteriosus
SLC19A1: Solute carrier family 19, member 1
SNP: Single nucleotide polymorphism
TGA: Transposition of the great arteries
TOF: Tetralogy of fallot
